# Integrating Nurse Preferences Into AI-Based Scheduling Systems: Qualitative Study

**DOI:** 10.2196/67747

**Published:** 2025-06-04

**Authors:** Fabienne Josefine Renggli, Maisa Gerlach, Jannic Stefan Bieri, Christoph Golz, Murat Sariyar

**Affiliations:** 1 School of Health Professions, Bern University of Applied Sciences, Bern, Switzerland; 2 School of Engineering and Computer Science, Bern University of Applied Sciences, Höheweg 80, Biel, 2502, Switzerland, 41 32 321 64 37

**Keywords:** nurse scheduling, work-life balance, feasibility, well-being, large language model, LLM, natural language processing, NLP, machine learning, ML, artificial intelligence, AI, AI-based scheduling, dissatisfaction, burnout, comprehensive framework, interview, mixed-integer programming, MIP, constraint programming, CP, reinforcement learning, RL

## Abstract

**Background:**

Nurse scheduling is a complex challenge in health care, impacting both patient care quality and nurse well-being. Traditional scheduling methods often fail to consider individual preferences, leading to dissatisfaction, burnout, and high turnover. Inadequate scheduling practices, including restricted autonomy and lack of transparency, can further reduce nurse morale and negatively affect patient outcomes. Research suggests that participative scheduling approaches incorporating nurse preferences can improve job satisfaction. Artificial intelligence (AI) and mathematical optimization methods, such as mixed-integer programming (MIP), constraint programming (CP), genetic programming (GP), and reinforcement learning (RL), offer potential solutions to optimize scheduling and address these challenges.

**Objective:**

This study aims to develop a framework for integrating nurses’ preferences into AI-supported scheduling methods by gathering qualitative insights from nurses and supervisors and mapping these to mathematical and AI-based scheduling techniques.

**Methods:**

Focus group interviews were conducted with 21 participants (nurses, supervisors, and temporary staff) from Swiss health care institutions to understand experiences and preferences related to staff scheduling. Qualitative data were analyzed using open and axial coding to extract key themes. These themes were then mapped to AI methodologies, including MIP, CP, GP, and RL, based on their suitability to address identified scheduling challenges.

**Results:**

The study revealed key priorities in nurse scheduling. Fairness and participation were highlighted by 85% (18/21) of interview participants, emphasizing the need for transparent and inclusive scheduling. Flexibility and autonomy were preferred by 76% (16/21), favoring shift swaps and self-scheduling. AI expectations were mixed: 62% (13/21) saw potential for improved efficiency and fairness, while 38% (8/21) expressed concerns over reliability and loss of human oversight. Mapping to AI methods showed MIP as effective for fair shift allocation, CP for complex rule-based conditions, GP for handling unforeseen absences, and RL for dynamic schedule adaptation in hospital environments. A preliminary AI implementation of MIP in a training hospital unit (35 staff members) showed how to design a system from a mathematical perspective.

**Conclusions:**

AI-supported scheduling systems can significantly enhance fairness, transparency, and efficiency in nurse scheduling. However, concerns regarding AI reliability, adaptability to individual needs, and human oversight must be addressed. A hybrid approach integrating AI recommendations with human decision-making may be optimal. Future research should explore the broader implementation of AI-driven scheduling models and assess their impact on nurse satisfaction and patient outcomes over time.

## Introduction

In the health care setting, nurse scheduling is a critical yet complex task that directly impacts both the quality of patient care and the well-being of nurses [1-4]. Traditional scheduling methods often fail to accommodate the preferences and constraints of individual nurses, potentially leading to dissatisfaction, burnout, and high turnover rates [5,6]. However, the extent of these negative effects and the specific factors contributing to dissatisfaction are often unclear. Nurses may feel restricted in their autonomy or, conversely, may desire more guidance to prevent feelings of unfairness [7]. In addition, issues may arise from either a lack of transparency in scheduling processes or an overload of information that leads to confusion [8]. Before considering improvements through formal or artificial intelligence (AI)–based scheduling methods, it is essential to thoroughly understand the current state and the specific needs and desires of the nursing staff. This allows then to map those needs to algorithmic scheduling solutions, ensuring an efficient transition to a desired future state that enhances both nurse satisfaction and patient care.

Several studies have examined health care workers’ preferences via interviews. Baljani et al [9] interviewed 20 managers and 2 clinical nurses and found that functional-exchangeable protection and flexible schedules reduce nurses’ family-related worries and work-family conflict, improving care quality and reducing errors. Booker et al [10] identified inadequate training on sleep and shift management, raising safety concerns. Saritas [11] interviewed 50 female nurses and showed that staffing shortages, intensified workloads, unpredictable schedules, and poor support worsen work-family conflict, making nurses reliant on spousal support and more likely to accept unfavorable conditions. Messing et al [12] reported that work-life balance issues relate to scheduling and family duties, with workers preferring stable schedules and consistent days off. Christopher [13] noted that only one-third of 26 interviewed nurse-mothers control their schedules: understaffing and long shifts compromise both patient and family care quality. Kim et al [14] identified key risk factors contributing to burnout syndrome, including work overload, demanding schedules, strained managerial relationships, poor work-life balance, and bureaucratic management.

Participative staff scheduling actively involves employees in creating work schedules, allowing them to express their preferences and needs regarding shifts and work hours. This approach enhances autonomy, flexibility, and job satisfaction by considering individual desires. Mrayyan et al [15] highlight that health care workers are often dissatisfied with limited autonomy, which is supported by management, education, and experience but undermined by authoritarian leadership, physicians, and high workload. Enhancing autonomy through targeted leadership and education is recommended. Christensen et al [16] illustrate how participatory management, using the Listen-Sort-Empower model, improves understanding of burnout causes like staffing shortages, work environment, and compensation, facilitating collaborative solutions. Similarly, Albargi et al [17] emphasize that empowering nurses in decision-making boosts engagement, job satisfaction, and patient outcomes. Shiri et al [18] found that digital participatory scheduling software improved employees’ control over their schedules, sleep quality, and perceived workability, but had no effect on psychological distress, self-rated health, or work-life conflict. Although participation of nurses involves more than just staff scheduling, these examples demonstrate that flexibility in this area is a crucial factor for overall job satisfaction.

Mathematical applications in participative staff scheduling leverage advanced techniques to optimize shift allocation based on employee preferences, availability, and operational requirements [19-23]. For stable shift planning incorporating preferences as constraints, 2 primary methods are used: mixed-integer programming (MIP) and constraint programming (CP). MIP addresses optimization problems with both continuous and discrete decision variables, making it suitable for scenarios where scheduling decisions must meet linear constraints and objectives [24]. It is effective for large-scale scheduling problems with well-defined constraints, such as total work hours, shift assignments, and resource allocation. MIP efficiently manages complex objective functions and constraints, including cost minimization and coverage maximization. Conversely, CP is designed for combinatorial problems involving complex, often nonlinear constraints. It focuses on feasibility rather than optimization and excels at managing diverse and intricate constraints and preferences [25]. It adeptly explores and refines the solution space to identify feasible schedules that meet all constraints.

Participatory staff scheduling can also be significantly enhanced by AI methodologies, especially by reinforcement learning (RL) and natural language processing (NLP). RL optimizes dynamic scheduling by adapting to evolving conditions through interaction and feedback, managing complex environments effectively [26-30]. NLP methods can process textual feedback from employees concerning their preferences and scheduling needs, converting it into actionable data for more personalized schedules, and with large language models even promise to provide solutions for the staff scheduling problem itself. In addition, heuristics like genetic algorithms complement MIP and CP, providing feasible solutions when exact methods to yield satisfactory results [31]. Furthermore, machine learning (ML) techniques, such as random forests, can be used to predict scheduling preferences based on historical data and patterns [32].

Currently, to our knowledge, there is no comprehensive framework that integrates nurses’ requirements for participatory shift scheduling within a broader set of methodologies. This paper aims to address this gap and present preliminary findings to facilitate broader implementation. The following section details the methodology for conducting interviews and how these results are mapped to the mathematical techniques. Subsequently, we will present the mapping result and initial outcomes of applying one of these mathematical methods, after which we conclude with a discussion of future perspectives. We adopt a high-level perspective and provide only a few illustrative mathematical formulas for clarity, as our primary audience includes nontechnical decision-makers.

## Methods

### Interviews

We conducted focus group interviews, for which the details and reporting checklist are provided in a paper still under review [33]. The study followed the COREQ (Consolidated Criteria for Reporting Qualitative Research) guideline to ensure comprehensive reporting. Participants were recruited through a convenience sampling approach, targeting permanent and temporary nursing staff from various health care settings, including acute hospitals, home care services, and nursing homes in German-speaking Switzerland. Recruitment flyers detailing the study’s purpose, procedure, and voluntary nature were distributed within participating institutions. Interested individuals contacted the research team directly to enroll in the study. The sample consisted of 21 participants, including nurse managers, permanent nurses, and temporary nurses, ensuring diverse perspectives on AI-based scheduling and work-life balance.

Here, we present just the summary. The interviews involved open-ended questions related to staff scheduling and prompt participants to articulate their thoughts, experiences, and opinions in their own words [34]. A total of 21 Swiss working staff participated in 4 focus group interviews. The central specific questions are presented in Table 1.

**Table 1.** Central interview questions by participant group in artificial intelligence (AI)–based nurse scheduling study. This table presents the key interview questions posed to different participant groups—supervisors, permanent nurses, and temporary staff—during qualitative focus group discussions conducted in Swiss health care institutions between May and June 2024. The study aimed to explore perceptions of existing scheduling systems, fairness, flexibility, and the potential role of AI-based solutions in nurse shift scheduling.

Interview group	Question
Supervisor	How does staff scheduling function as a management tool in your department? How do you manage staff absences and ensure fairness in scheduling? How does scheduling impact staff satisfaction? What features do you expect from an AI-based scheduling system?
Nurses	How well does the current scheduling system meet your needs for time off and work-life balance? What scheduling challenges do you face in hospital and outpatient care settings? How do shift lengths and scheduling practices affect your job satisfaction and recovery? What improvements would enhance your scheduling experience?
Temporary staff	How does flexibility in creating your own schedule compare with that of permanent staff? How do you balance personal scheduling needs with institutional requirements?

### Ethical Considerations

#### Human Participants Research Ethics Review, Exemptions, and Approvals

This study was conducted in accordance with the ethical guidelines of the Bern University of Applied Sciences. The local ethics committee confirmed that the study did not require a full ethical review and was not covered by the Swiss Federal Law on research involving humans. As such, a formal institutional review board approval was not necessary. However, all research activities adhered to principles of ethical research, ensuring respect for participants’ rights and well-being.

#### Informed Consent

Before participation, all individuals were provided with a detailed study information sheet explaining the research purpose, methodology, potential risks, and benefits. Participants voluntarily provided written informed consent before their involvement in the study. As this study involved secondary qualitative analysis of interview data, all primary data were collected with consent that permitted subsequent secondary analysis.

#### Privacy and Confidentiality Protection

To protect participant confidentiality, all interview data were anonymized before analysis. Identifying information, such as names, job titles, and specific workplace locations, was removed or altered to prevent reidentification. Data storage was secured in password-protected files, accessible only to authorized research team members. No personally identifiable images or information were included in the study.

#### Compensation

No financial or material compensation was provided to participants for their involvement in this study. Participation was entirely voluntary, and no incentives were offered to ensure unbiased responses.

#### Protection of Individual Identity in Images

No images or supplementary materials in the manuscript contain identifying features of participants. In the event that identifiable images were deemed necessary, written informed consent would be obtained, and the appropriate consent documentation would be provided upon submission. However, as this study relies solely on anonymized qualitative data, no such images are included.

### Mapping Requirements to Methods

The mapping process occurs in two stages: (1) deriving key themes from the interview summaries, and (2) the actual mapping, where relevant AI methods are assigned based on the identified categories to address the specific needs of the interview participants. In stage 1, key themes from the interviews were extracted and axial coding used to organize these themes into overarching categories [35]. Subsequently, expectation and concerns regarding AI in this context is summarized. In stage 2, of the mapping process, we align the categories identified during the interviews with appropriate AI methodologies. Coauthor MS, who possesses the necessary technical expertise, provides the rationale for selecting the most suitable AI methods–MIP, CP, GP (genetic programming [36]), and RL–to address each identified need. For one category identified as feasible for immediate implementation, an exemplary implementation is shown. Table 2 outlines the key characteristics of these methods.

**Table 2.** Artificial intelligence (AI) methods and their properties for nurse scheduling optimization. This table provides an overview of 4 AI-based scheduling techniques, and their suitability for optimizing nurse shift scheduling.

Method	Properties
MIP^a^	Optimization: Finds the best solution from a range of possible options, conditioned by linear constraints. Modeling: Can integrate complex constraints and objectives. Computational demand: Can be computationally intensive for large problem sizes. Flexibility: Adaptable to various scheduling scenarios.
CP^b^	Focus on constraints: Solves problems through logical conditions and constraint propagation. Modeling: Suitable for problems with many constraints. Flexibility: Allows for complex and dynamic constraints. Solution space: Effective in searching for solutions in large and complex solution spaces.
GP^c^	Search method: Uses evolutionary theories to find solutions. Adaptability: Can adjust to different problem types. Heuristic: Provides good solutions by exploring the solution space but does not guarantee the optimal solution. Application: Well-suited for problems with many variables and nonlinear constraints that can rather be tested than modeled.
RL^d^	Learning approach: Learns through rewards and penalties from interactions with the environment. Adaptability: Can adjust to changes in the environment and requirements. Short-term planning: Suitable for dynamic and evolving scheduling tasks. Complexity: Requires a lengthy training phase and extensive data for effective learning.

a MIP: mixed-integer programming.

b CP: constraint programming.

c GP: genetic programming.

d RL: reinforcement learning.

A few words on RL, it has not been widely applied to personnel scheduling so far. However, it holds great promise, as traditional scheduling methods often struggle to accommodate the numerous constraints and sudden changes inherent in such environments. RL offers a dynamic and adaptive approach to these challenges. It can be particularly useful in three key areas: (1) optimizing shift exchanges: RL can learn effective strategies for reallocating shifts to improve overall schedule quality while respecting operational constraints. (2) Dynamic and ad-hoc rescheduling: When unforeseen absences or sudden demand fluctuations occur, RL can adjust schedules in real-time to maintain adequate staffing levels. And (3) personalized scheduling across life stages: by considering individual work preferences and constraints, RL can help create schedules that balance organizational needs with personal well-being.

By integrating RL into scheduling systems, health care institutions could move toward more flexible, efficient, and fair staff allocation methods. While its application in this field is still in its early stages, its potential to enhance nurse scheduling and workforce management is clear.

## Results

### Key Themes From the Interviews and Expectations Regarding AI

A consistent theme across interviews is the demand for fair and participative staff scheduling. Participants stress that in health care environments, it is crucial to include staff in scheduling decisions, consider individual preferences, and maintain transparency. These elements are seen as foundational for promoting satisfaction and efficiency among staff. Stability in shift assignments, particularly on weekends, is important to many. In addition, the ability to swap shifts within teams is viewed as essential for flexibility. Fairness, in this context, implies equal treatment for all employees and the opportunity to contribute to schedule planning, particularly regarding weekend shifts. Transparent and clear communication is identified as vital for fostering trust in the scheduling process. In outpatient care settings, participative scheduling extends to balancing workload and start times to ensure fairness. For supervisors, achieving a balanced mix of patient cases is critical to prevent specific employees from being consistently assigned complex cases. Overall, the analysis identified five key themes reflected in the summaries, which contribute to overall dissatisfaction and the desire for changes: (1) importance of scheduling as a management tool: staff scheduling is viewed as a critical element in effective management, particularly in structured and high-demand settings like intensive care units; (2) challenges in maintaining fairness: despite established frameworks, ensuring fairness in scheduling is difficult due to varying circumstances and competing needs; (3) complexity in managing absences: handling short-notice absences often relies on informal or ad-hoc methods, highlighting gaps in systematic approaches; (4) flexibility in outpatient care: the dynamic nature of outpatient care, driven by client turnover, necessitates greater flexibility from staff and poses unique scheduling challenges; and (5) impact on work-life balance and employee morale: scheduling decisions directly affect employees’ personal lives, often triggering emotional responses, making fairness and transparency critical but difficult to maintain.

In addition to that, the interviewees formulated expectations regarding AI, especially for streamlining the scheduling processes. AI is expected to handle a wide array of individual requirements and preferences, such as avoiding late shifts on specific days or adjusting work schedules based on partial employment percentages. The AI system should also accommodate experience levels and qualifications, manage fluctuating monthly and annual work hours, and efficiently address overtime. In addition, AI should consider nonclinical responsibilities, such as supervisory duties or mentoring, which are often overlooked in traditional scheduling. For outpatient care, it is critical that AI factors in the varying demands of different clients and incorporates lunch breaks into schedules.

Participants also expect AI to serve as a neutral entity that can enhance team dynamics. A well-configured AI system could act as a neutral arbiter, reducing emotional tensions that often arise during scheduling discussions. Ideally, the system would provide a baseline schedule that can be adjusted as needed, considering recent (potentially unfair) scheduling trends and distributing clients more equitably. Participants highlight the potential for increased scheduling accuracy, improved efficiency, and a reduction in both administrative workload and scheduling errors. These improvements could result in more time for patient care, increased employee satisfaction, and enhanced working conditions characterized by transparency and fairness.

Despite the potential advantages of AI-based scheduling, several significant concerns must be addressed. Achieving perfect accommodation for every individual’s requirement is nearly impossible, and limitations in the system’s ability to address all requests could result in dissatisfaction. Reliability of AI systems is another critical concern. There is apprehension about overreliance on technology, which could lead to operational disruptions in the event of system failures. Furthermore, AI systems depend on the quality of the data they process. Ensuring that this data is accurate and up to date requires ongoing vigilance, presenting an additional challenge. However, this situation also represents an opportunity for digital transformation. Addressing such concerns proactively could improve overall system effectiveness.

### Map Between Key Issues and AI Methods

The mapping presented in Table 3 ensures that each key thematic issue is addressed using a method that best meets its unique requirements and challenges. This does not imply that a combination of methods is always necessary in practice. On the one hand, the theoretically most promising approach must also demonstrate practical success, on the other hand, methods such as RL can address many issues autonomously. Therefore, the final choice of mapping depends on context-specific conditions. Our experience suggests that maintaining a repertoire of methods is advantageous, as ensemble-based decisions can enhance validity and objectivity. Relying solely on a single method should be approached with caution, even if it can address numerous issues independently.

**Table 3.** Artificial intelligence (AI)–based optimization methods for key staff scheduling challenges. This table presents the most suitable AI methods for resolving critical nurse scheduling challenges identified in Swiss health care institutions between may and June 2024.

Key issue	Most suitable method	Reasoning
Scheduling as a management tool	MIP^a^	MIP is well-suited for optimizing complex scheduling scenarios with defined constraints and objectives, ensuring effective management in structured settings like ICUs^b^
Maintaining fairness	CP^c^	CP focuses on constraints and can handle complex conditions, making it suitable for ensuring fairness and accommodating various needs within scheduling
Managing absences	GP^d^	GP’s heuristic approach and adaptability make it effective for handling unpredictable issues such as unplanned absences, exploring various solutions. RL^e^ would also be suitable
Flexibility in outpatient care	RL	RL can automatically adjust to dynamic changes and evolving requirements, making it ideal for the flexible and changing nature of outpatient care scheduling.
Work-life balance and employee morale	MIP	MIP’s optimization capabilities can help balance employee needs and scheduling constraints, addressing work-life balance while ensuring fairness and efficiency. Methodologically addressed through penalty terms for violating constraints.

a MIP: mixed-integer programming.

b ICU: intensive care unit.

c CP: constraint programming.

d GP: genetic programming.

e RL: reinforcement learning.

For 2 key issues, MIP emerged as the most suitable method. This is not surprising given that MIP is supported by well-established heuristics such as branch-and-bound [37] and cutting-plane methods [38], which have long been validated for solving complex problems. MIP can also address work-life balance and employee morale by optimizing schedules with penalty terms for constraint violations. However, there are complex scenarios where MIP must be replaced by methods such as GP or RL. This limitation may arise either because the overall constraints are infeasible, a common issue in practice, or because the constraints are too complex for MIP. For these reasons, we recommend maintaining a diverse set of methods. CP is suitable for maintaining fairness by handling complex conditions. GP proves effective for handling unpredictable absences due to its heuristic nature, which facilitates rapid adaptation to changes in existing constraints. In contrast, RL is particularly suited to dynamically changing outpatient care environments, as it allows for the seamless integration of novel constraints.

### Implementation of a Mathematical Method

In a specific scenario, we apply MIP to optimize staff scheduling for a training unit in a hospital. The scheduling model covers a representative week. The staff comprises 35 employees categorized into 4 types: 2 doctors, 3 experienced nurses (Type A), 20 moderately experienced nurses (Type B), and 10 less experienced nurses (Type C). Due to the integration of training and care responsibilities, the scheduling involves complex requirements, as most nurses are in training. There are 2 shifts per day, and each employee can either work or not work these shifts. Scheduling is done based on employee type and availability. Each employee is allowed to work a maximum of 6 days per week, with a minimum requirement of 4 days for all employees except doctors. Since doctors have fixed working hours, they are excluded from the scheduling model.

First, with regard to staffing levels per shift, each shift (morning and evening) must consist of at least 3 employees. Specifically, the morning shift must include a minimum of 8 employees, while the evening shift must have at least 3 employees. Second, with regard to specific shift requirements, the morning shift must include at least 3 nurses of types A or B. The morning shift must also include at least 2 nurses of type C. The evening shift must include at least 2 nurses of types A or B. The evening shift must include at least 1 nurse of type C. Third, with regard to external constraints, trainees (under 18 y old) are not permitted to work on Sundays. Employees with school obligations are not allowed to work on the days they have classes. Fourth, with regard to preferred shifts: Employee preferences for specific shifts and days must be taken into account. Shifts that do not align with employee preferences will incur a higher penalty in the objective function.

To formulate these conditions mathematically, we define binary variables $x\left[i,j,k\right]$, which indicate whether person *i* works on day *j* during shift *k*, as follows: *i* = 1,..., 33 (employees); *j* = 1,...7 (days in the week); *k* = 1, 2 (*k*=1 represents the morning shift, and *k*=2 represents the evening shift).

Each variable $x\left[i,j,k\right]$ takes values in {0,1}, where 1 means that employee i is assigned to shift k on day j, and 0 otherwise. On this basis, conditions can be established. For example, to ensure that each employee works at least 4 days per week, the constraint is formulated as follows:


$$\sum_{j=1}^{7}\sum_{k=1}^{2}x\left[i,j,k\right]\geq 4,i=1,\ldots 33$$

Instead of directly integrating employee types into the indices of binary variables, employee types are modeled through separate variables and then multiplied by the existing binary variables to define constraints. This approach simplifies the modeling and comprehension of constraints and enhances flexibility, as employee types can be reassigned without necessitating changes to the constraints. Further conditions are omitted here for brevity (for details, refer to [35]).

The R package (R Core Team) OMPR was used to solve this problem [39]. The solution requires at least 8 personnel per shift. Given that each employee is scheduled to work at least 4 days, the staffing counts per day and shift reveal inefficiencies: some shifts have up to 20 employees, which is suboptimal. By reducing the minimum number of working days from 4 to 2, a more efficient schedule is achieved. Although this is a training station, and the inefficiency is not critical, it demonstrates that with numerous constraints, even linear ones, inefficiencies can arise rapidly.

We also trained a graph neural network-based RL model for shift assignments, using one million epochs and batch sizes of 1280 to optimize scheduling. However, RL underperformed compared to MIP, failing to converge despite extensive training. This outcome underscores the challenges of applying neural networks to personnel scheduling, where high-dimensional constraints and sparse reward signals can impede learning stability.

## Discussion

### Principal Findings

The summary of the interviews showed a strong demand for fair and participative staff scheduling within health care environments. Interview participants emphasized the importance of including staff in scheduling decisions, considering individual preferences, and ensuring transparency. These elements are seen as key drivers for job satisfaction and operational efficiency. Fairness in scheduling, particularly around weekend shifts, was a recurring concern, with many advocating for equal opportunities in contributing to the schedule planning process. The ability to swap shifts within teams and balancing workload distribution were identified as critical factors for maintaining flexibility. In outpatient care, there was also an emphasis on adjusting start times and distributing patient cases evenly to prevent any single employee from consistently handling more complex cases. Overall, five key challenges emerged: scheduling as a management tool, maintaining fairness, managing absences, ensuring flexibility in outpatient care, and their collective impact on work-life balance and employee morale.

In addition to traditional scheduling expectations, participants expressed a clear interest in integrating AI to streamline processes. AI was viewed as a potential solution for managing complex individual requirements, such as avoiding late shifts or adjusting schedules based on employment percentages and qualifications. Furthermore, AI was expected to accommodate fluctuating work hours, account for nonclinical responsibilities like mentoring, and improve equity in client case distribution. There was optimism that AI could serve as a neutral entity, reducing emotional tensions and providing an initial schedule that could be adjusted as needed. However, concerns were raised about the reliability of AI systems, potential loss of human elements in decision-making, and the importance of maintaining supervisor oversight to ensure flexibility and contextual understanding.

The study’s mapping of key scheduling issues to AI methods highlights the importance of aligning specific challenges with appropriate technological solutions. Each identified key issue requires distinct approaches to optimize scheduling processes. For instance, MIP is deemed most suitable for managing structured, complex scheduling scenarios with defined constraints. CP excels in addressing fairness by focusing on complex constraints and balancing competing needs. GP is selected for its heuristic approach to handling highly complex constraints, while RL offers the adaptability needed for the dynamic nature of staff scheduling. This method-specific approach ensures that each challenge is addressed using the most effective technique, potentially enhancing scheduling efficiency and overall staff satisfaction. Maintaining a repertoire of methods allows for a more flexible and comprehensive solution and mutual validation, acknowledging that no single method is universally applicable.

Our results also indicate the challenges of applying RL to nurse scheduling. While RL offers adaptability, our graph neural network-based RL model struggled to converge despite extensive training, likely due to high-dimensional constraints and sparse reward signals. Future improvements, such as enhanced reward shaping, constraint-aware RL architectures, or hybrid approaches combining RL with rule-based methods, may be necessary to improve RL’s effectiveness in this complex domain. By integrating RL’s adaptability with the precision of optimization techniques, future research could refine AI-driven scheduling for dynamic health care environments.

### Comparison With Previous Work

This study aligns with previous research emphasizing the complexity and challenges of staff scheduling in health care settings [40]. Similar studies have highlighted the difficulties in balancing fairness, flexibility, and operational efficiency [41]. By offering a challenge-based mapping of the most pertinent methodologies, this study advances the discourse surrounding staff scheduling, emphasizing the importance of a comprehensive and integrative approach.

### Limitations

This study has several limitations. First, the reliance on interviews for data collection may introduce subjective biases and limit the generalizability of findings. Nurses and supervisors may have different perspectives that are not fully captured through qualitative interviews alone. In addition, the study’s focus on specific hospital and outpatient care settings may not fully represent the diverse range of health care environments, potentially limiting the applicability of findings to other contexts.

Another limitation lies in the fact that the mapping between key issues and AI methods was based on the expertise of one expert, rather than being grounded in a more objective, data-driven approach. Achieving a comprehensive, objective mapping would require extensive knowledge, not only of the diverse AI techniques but also of the practical demands of health care environments. This would necessitate a significant collaborative effort across multiple disciplines, including ML, optimization, human resource management, and health care.

We did not specifically address how to accurately capture employees’ preferences. Often, individuals may lack full awareness of their own desires or what would be most beneficial to them. However, this raises a sensitive issue, as it involves the risk of paternalism. Not every form of objectification necessarily enhances well-being, given that autonomy is a highly valued principle. In a separate context, we are working on a system that seeks to balance objectification and autonomy in the process of eliciting preferences.

Furthermore, the study acknowledges the limitations of AI in addressing the emotional and psychological states of employees. While AI can improve scheduling efficiency, it lacks the sensitivity needed to manage complex human factors, which could negatively impact the overall effectiveness of the scheduling process. This limitation highlights broader concerns about the role of AI in decision-making processes that involve significant human interaction and emotional considerations.

### Conclusions

AI-based scheduling presents a promising solution, offering potential benefits such as increased accuracy, efficiency, and reduced administrative workload. However, it is essential to address concerns related to the limitations of AI, including its ability to fully accommodate individual preferences and the risk of overreliance on technology. A balanced approach that incorporates both advanced scheduling methods and human oversight is crucial to achieving optimal outcomes. Future research should investigate ways to enhance the integration of AI with human judgment to address the emotional and psychological aspects of scheduling. Finally, it is worthwhile to investigate whether large language models are capable of independently conducting staff scheduling [42].

## Abbreviations

AI: artificial intelligence
COREQ: Consolidated Criteria for Reporting Qualitative Research
CP: constraint programming
GP: genetic programming
MIP: mixed-integer programming
ML: machine learning
RL: reinforcement learning
